# Validity of the GAITRite Walkway Compared to Functional Balance Tests for Fall Risk Assessment in Geriatric Outpatients

**DOI:** 10.3390/geriatrics5040077

**Published:** 2020-10-17

**Authors:** Johannes Riis, Stephanie M. Byrgesen, Kristian H. Kragholm, Marianne M. Mørch, Dorte Melgaard

**Affiliations:** 1 Center for Clinical Research, North Denmark Regional Hospital, 9800 Hjørring, Denmark; s.byrgesen@rn.dk (S.M.B.); kdks@rn.dk (K.H.K.); dmk@rn.dk (D.M.); 2 Department of Geriatric Medicine, North Denmark Regional Hospital, 9800 Hjørring, Denmark; m.moerch@rn.dk; 3 Department of Clinical Medicine, Aalborg University, 9000 Aalborg, Denmark

**Keywords:** falls, GAITRite, concurrent validity, walking aids

## Abstract

This study examined the concurrent validity between gait parameters from the GAITRite walkway and functional balance test commonly used in fall risk assessment. Patients were sampled from one geriatric outpatient clinic. One physiotherapist evaluated the patients on the GAITRite walkway with three repetitions in both single- and dual-task conditions. Patients were further evaluated with Bergs Balance scale (BBS), Dynamic Gait index (DGI), Timed Up and Go (TUG), and Sit To Stand test (STS). Correlations between quantitative gait parameters and functional balance test were analyzed with Spearman’s rank correlations. Correlations strength was considered as follows: negligible <0.1, weak 0.10–0.39, moderate 0.40–0.69, and strong ≥0.70. We included 24 geriatric outpatients in the study with a mean age of 80.6 years (SD: 5.9). Patients received eight (SD: 4.5) different medications on average, and seven (29.2%) patients used walkers during ambulation. Correlations between quantitative gait parameters and functional balance test ranged from weak to moderate in both single- and dual-task conditions. Moderate correlations were observed for DGI, TUG, and BBS, while STS showed weak correlations with all GAITRite parameters. For outpatients analyzed on the GAITRite while using walkers, correlations showed no clear pattern across parameters with large variation within balance tests.

## 1. Introduction

The world’s population is aging as the proportion of older adults is increasing, while in the western world, 22.1% of the population is expected to be above 65 years of age by the year of 2030 [1]. With the population aging, age-related conditions such as falls and problems related to mobility will increase in frequency [1]. About 33% of adults over 65 years of age experience a fall each year, and this proportion increases further with increasing age [2,3]. Falls in older adults lead to serious consequences, such as excess mortality, morbidity and loss of independence [4]. Furthermore, falls are also associated with great economic strain on health care systems [5].

In order to prevent falls, older adults who experience more than one fall in a year or who show problems related to gait and mobility should receive examination and fall risk assessment [3,4,6]. This is commonly done in geriatric outpatient clinics, and the assessments often include multiple functional balance tests to ascertain the risk of future falls [7,8]. As more patients may require assessment in the future, and as the patient group is physically and cognitively frail and may not be able to undergo long and demanding examinations in one consultation, there is a need for implementation of effective tools for fall risk assessment.

Instrumented walkways are among new technologies used in gait assessment. One of the most frequently used instrumented walkways is the GAITRite walkway, which also has the advantage that patients can be tested while using walking aids [9]. The GAITRite walkway allows for a reliable and valid measurement of spatiotemporal gait parameters [10,11], which has been shown to be useful for predicting falls [9,12,13]. However, studies examining the convergent validity of this walkway compared to functional balance tests in the setting of fall risk assessment are sparse, and, therefore, we chose to examine this in a sample of geriatric outpatients.

## 2. Materials and Methods

We included consecutive geriatric outpatients referred due to problems related to mobility (most often falls) seen at the Regional Hospital of Northern Jutland between December 2018 and March 2019. The present study was registered with the Danish Data Protection Authority (2008-58-0028). The local Ethics Committee waived the need for written informed consent.

Patients were assessed by a multidisciplinary team that included a physiotherapist and a medical doctor. The medical doctor (registrar or consultant in geriatric medicine) performed a comprehensive geriatric assessment, including a review of the patients’ medication and clinical examination for underlying medical conditions affecting gait, such as peripheral polyneuropathy. The physiotherapist assessed the patients using several functional balance tests as well as the GAITRite walkway (CIR Systems, Inc., 12 Cork Hill Rd, BLDG 2, Franklin, NJ, USA). All patients were seen by the same physiotherapist, and test were administered in the same order. A total of 45 min was set aside for the physiotherapist examination. Patients who displayed signs of cognitive impairment were also tested using the Mini-Mental State Examination.

The functional balance tests that patients received were the Bergs Balance Scale (BBS), Dynamic Gait Index (DGI), Timed Up and Go (TUG), and Sit To Stand test (STS). These tests are commonly used to assess patients dynamic balance and to give an assessment of patients’ risk of future falls [7,8].

Patients were then tested on the GAITRite walkway during both single- and dual-task conditions. They were tested on the walkway three times for each condition. They were instructed to walk on the walkway in their usual pace, and if patients were reliant on walking aids such as walkers for daily ambulation, they were tested using these. For the dual-task condition, patients were instructed to count backwards from 50 while completing the walks in the usual pace.

Data from the three walks on the GAITRite walkway were analyzed automatically using the GAITRite software (version 4.8.7, Franklin, NJ, USA) for each condition. In cases where footfalls or walking aids were not correctly identified automatically, this was corrected manually. Based on the three walks for each condition, the software calculated the following parameters, which were analyzed in this study: velocity, cadence, stride length, swing phase time, double support time, stride length variability, and swing phase time variability. Variability was calculated by coefficients of variability of the parameters.

We collected further variables from the patients’ medical records. Here, diagnoses including new diagnoses given following comprehensive geriatric assessment were coded in patients’ medical records according to the international classifications of diseases version 10 (ICD-10). Information on medication at the time of assessment was also available from the medical records, and from this, we gathered information on the total number of medications a patient was taking at the time, and whether or not patients were taking drugs associated with increased fall risk, such as opioids, alpha-blockers, anticholinergic agents, antihistaminergic agents, benzodiazepines, or antidepressants. All patient also had a social history taken, and from this, we gathered data on whether patients resided at home or in nursing homes, and if they resided at home, whether they received home health care or were independent in daily living. We also registered if patients were living alone or not.

Patient characteristics were presented as numbers and percentages in the case of categorical variables and as means and standard deviations in the case of continuous variables. Normality of continuous variables was assessed by visual inspection of histograms, and in the case of non-normal distributions, variables were instead presented as medians and interquartile range. We examined the correlation between GAITRite parameters and the functional balance tests using unadjusted Spearman’s rank correlation coefficients. These were presented visually by using a correlation plot, where color saturation and dot size represented the correlation strength. We also calculated the average of the absolute correlation values across all functional balance test during both single- and dual-task conditions for each GAITRite parameter to examine which parameter had the strongest and weakest correlations with the functional balance tests. The strength of correlations based on numeric values were considered as follows: negligible < 0.1, weak 0.10–0.39, moderate 0.40–0.69, and strong ≥ 0.70 [14]. Finally, we analyzed the same correlations separately in the subgroup of patients who were tested while using walkers to see if this affected the results.

## 3. Results

### 3.1. Patient Characteristics

We included 24 geriatric outpatients in the study period (Table 1). The mean age of included patients was 80.6 years, and 15 (62.5%) were female. Patients were most often referred due to a history of falls (75%), and only one patient resided in a nursing home.

### 3.2. Correlation between GAITRite Paramters and Functional Balance Tests in All Patients

**Figure 1:**
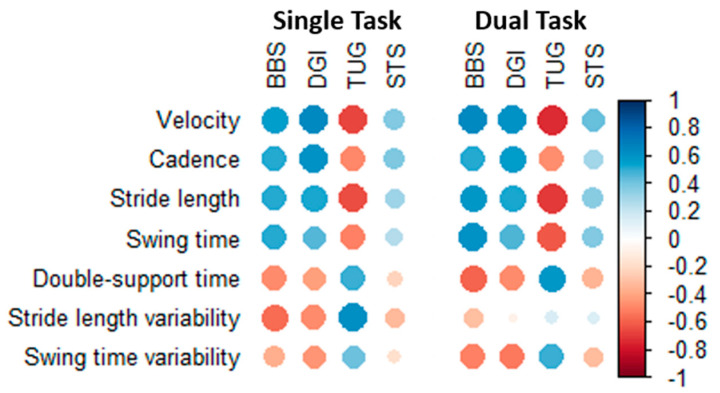
Correlation between GAITRite parameters and functional balance tests in all patients. Blue represents positive correlations, and red represent negative correlations. Color saturation and dot sizes represent correlation strength. Abbreviations: BBS, Bergs Balance Scale; DGI, Dynamic Gait Index; TUG, Timed Up and Go; STS, Sit To Stand test.

All GAITRite parameters showed weak to moderate correlation with the functional balance test both in single- and dual-task conditions (Figure 1, values available in Table S1). Correlations with BBS, DGI, and TUG were moderate for most GAITRite parameters, while correlations with STS were weak. Velocity and stride length were most correlated with the functional balance test on average, while stride length variability and swing time variability showed the weakest correlations with the functional balance test (Table S1).

### 3.3. Correlation between GAITRite Paramters and Functional Balance Tests in Walker User

**Figure 2:**
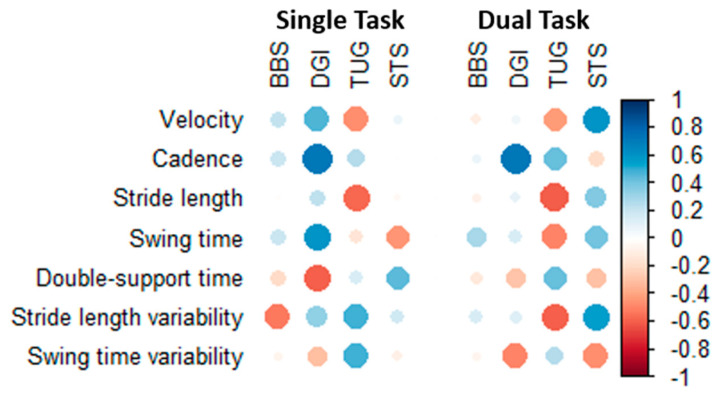
Correlation between GAITRite parameters and functional balance tests in patients using walkers. Blue represents positive correlations, and red color represent negative correlations. Color saturation and dot sizes represent correlation strength. Abbreviations: BBS, Bergs Balance Scale; DGI, Dynamic Gait Index; TUG, Timed Up and Go; STS, Sit To Stand test.

When analyzing correlations separately in the seven patients tested while using walkers, correlation results appeared random with large variations in results within the same balance test and did not appear to follow the same patterns as for the entire sample (Figure 2, values in Table S2). Results when analyzing correlations in the remaining patients not using walkers were similar to the primary results (Figure S1, values in Table S3).

## 4. Discussion

This study reports the convergent validity of the GAITRite walkway in dual- and single-task conditions compared to functional balance tests in geriatric outpatients. We showed that most GAITRite parameters showed moderate correlations with BBS, DGI, and TUG, while they showed weak correlations with STS. Finally, we showed that the correlation in patient tests using walkers had a random pattern and did not follow the same pattern as in the entire patient sample.

We found that GAITRite parameters were moderately correlated with DGI and TUG, which is unsurprising as both balance tests have a substantial component assessing gait [15]. The fact that the results are similar for BBS may seem more surprising, as this test primarily assesses dynamic balance in stance and postural change; however, it has previously been shown that there is a moderate correlation between BBS and DGI in patients with vestibular dysfunction, and our findings for fall risk assessment in geriatric outpatients are in line with this [16]. Finally, we showed weak correlations between GAITRite parameters and STS, which is in line with STS primarily being a measure of change in posture and not gait, and our correlation strength is similar to the results of a previous study comparing a sit to stand test with a walking test [17].

We are among the first to examine the convergent validity of the GAITRite walkway compared with functional balance tests in the setting of fall risk assessment. One study compared the Tinetti gait section score with the GAITRite results in a patient with normal pressure hydrocephalus, and strong correlations between the two tests were found, while the GAITRite walkway seemed to be the best of the two in discriminating gait alterations [18]. Compared to the functional balance tests used in our study, the Tinetti gait section score is focused on subjective assessment of gait characteristics very similar to those measured by the GAITRIte walkway, confirming our results that positive correlations between GAITRite and functional balance tests are more pronounced in tests with a significant gait component.

Another study compared GAITRite parameters with results of modified BBS in patients with dementia [19]. They found results similar or stronger than those in this study, which supports our findings. However, they found that stride length variability and swing time variability were most highly correlated with the modified BBS, which is in contrast with our results, where these parameters were generally the least correlated with functional balance tests. These differences may be due to dementia affecting stride length and swing time variability more than overall performance, which is also strengthened by the fact that these correlations were most affected when the authors adjusted for Mini-Mental State Examination (MMSE) and age.

In the context of assessing the risk of future falls, the correlation strengths found in this study are not strong enough to allow for GAITRite parameters to replace any of the functional balance tests. Nevertheless, the GAITRite walkway may add supplemental information that is useful in the overall assessment, as it allows for quantitative measurement of gait parameters that are not easily measured otherwise and could be used in monitoring effects of clinical interventions. Furthermore, the GAITRite walkway can assist in standardized diagnosis of gait abnormalities, which is otherwise highly dependent on examiner expertise [20]. Previous studies have indicated that GAITRite parameters (including all GAITRite parameters measured in the present study) are predictive of incident falls and fall history, and may therefore provide useful prognostic information with assessing fall risk [9,12,13,19,20], but further studies should determine whether GAITRite parameters still add predictive information when taking into account results of functional balance tests.

Finally, we found no clear correlation between GAITRite parameters and functional balance tests in patients tested while using walkers. A previous study compared GAITRite parameters between patients using walkers and patients walking without walkers, and different gait patterns in patients using walkers were found, which may explain the lack of correlation found in our study [9]. They also found higher risk of incident falls in patients using walkers, which is most likely due to underlying medical conditions, as walkers are generally thought to be protective against falls [21]. Whether or not spatiotemporal gait parameters are still predictive of falls in these patients remains unknown and should be investigated in further studies.

Our study has some limitations. First, our study is based on a small sample of patients, and, therefore, the results should be verified in a larger study. Due to the sample size, we abstained from regression analysis, which could potentially provide information of value in a larger study. This is especially true for the results regarding patient using walkers. Furthermore, the included sample is very heterogeneous. This is representative of the target population of geriatric patients but can result in larger statistical variation in the results. Second, there are some missing data regarding cognitive function and smoking status of patients, which may make interpretation and generalization of our results harder. Finally, a strength is that our study is based on real patients from a geriatric outpatient clinic. This strengthens the generalizability of our results to clinical practice, but the heterogeneous nature of a geriatric patient sample may reduce the correlation strengths found in this study when compared to more selective patient groups.

## 5. Conclusions

Correlations between quantitative gait parameters and functional balance test ranged from weak to moderate in both single- and dual-task conditions. For patients tested on the GAITRite while using walkers, correlations showed no clear pattern across parameters with large variation within balance tests.

## Figures and Tables

**Table 1:** Characteristics of included patients

Characteristic	Total *n* = 24
Age—mean ± SD	80.6 ± 5.9
Female sex—*n* (%)	15 (62.5%)
BMI—mean ± SD	26.6 ± 3.6
Missing	2
Smoking—current/prior	10 (76.9%)
Missing	11
Falls in previous year—*n* (%)	18 (75.0%)
Peripheral polyneuropathy—*n* (%)	9 (37.5%)
History of heart disease—*n* (%)	6 (25.0%)
History of stroke—*n* (%)	6 (25.0%)
Hypertension—*n* (%)	17 (70.8%)
Diabetes—*n* (%)	4 (16.7%)
COPD—*n* (%)	2 (8.3%)
Walker use—*n* (%)	7 (29.2%)
No. of drugs—mean ± SD	8 ± 4.5
Use of FRIDs—*n* (%)	12 (50.0%)
Living alone—*n* (%) ^1^	11 (47.8%)
Home health care recipient—*n* (%) ^1^	10 (43.5%)
MMSE—median (IQR)	24.5 (19.5, 28.0)
Missing	16

^1^ Excluding one patient residing in a nursing home. Abbreviations: COPD, Chronic Obstructive Pulmonary Disease; FRIDs, Fall Risk Increasing Drugs; MMSE, Mini-Mental State Examination; IQR, Interquartile Range; BMI, Body Mass Index.

## Supplementary Materials

The following are available online at https://www.mdpi.com/2308-3417/5/4/77/s1, Figure S1: Correlation between GAITRite parameters and functional balance tests in patients not using walkers. Blue represents positive correlations, and red represents negative correlations. Color saturation and dot sizes represent correlation strength. Abbreviations: BBS, Bergs Balance Scale; DGI, Dynamic Gait Index; TUG, Timed Up and Go; STS, Sit To Stand test. Table S1: Correlation values between GAITRite parameters and functional balance tests in all patients. Abbreviations: BBS, Bergs Balance Scale; DGI, Dynamic Gait Index; TUG, Timed Up and Go; STS, Sit To Stand test. Table S2: Correlation values between GAITRite parameters and functional balance tests in patients using walkers. Abbreviations: BBS, Bergs Balance Scale; DGI, Dynamic Gait Index; TUG, Timed Up and Go; STS, Sit To Stand test. Table S3: Correlation values between GAITRite parameters and functional balance tests in patients not using walkers. Abbreviations: BBS, Bergs Balance Scale; DGI, Dynamic Gait Index; TUG, Timed Up and Go; STS, Sit To Stand test.
