# Supplementary material for: Validity of the GAITRite Walkway Compared to Functional Balance Tests for Fall Risk Assessment in Geriatric Outpatients
Source: Geriatrics (Basel). 2020 Oct 17;5(4):77. doi: 10.3390/geriatrics5040077 (PMC7720110; doi:10.3390/geriatrics5040077)
Supplement: Supplementary file 1 [file geriatrics-05-00077-s001.zip › supplementary/Table S1-3.pdf]

Table S1. Correlation between GAITRite parameters and functional balance tests in all patients.

	Singe task				Dual task				Average
	**BBS**	**DGI**	**TUG**	**STS**	**BBS**	**DGI**	**TUG**	**STS**	**(absolute)**
Velocity	0.55	0.64	-0.67	0.37	0.64	0.61	-0.73	0.42	0.58
Cadence	0.52	0.60	-0.48	0.38	0.52	0.57	-0.47	0.30	0.48
Stride length	0.51	0.53	-0.64	0.31	0.58	0.53	-0.69	0.36	0.52
Swing time	0.51	0.45	-0.51	0.26	0.60	0.47	-0.62	0.38	0.48
Double-support time	-0.48	-0.41	0.49	-0.22	-0.59	-0.47	0.58	-0.35	0.45
Stride length variability	-0.56	-0.47	0.62	-0.33	-0.31	-0.07	0.15	0.13	0.33
Swing time viarability	-0.36	-0.44	0.41	-0.18	-0.50	-0.52	0.49	-0.31	0.40

Abbreviations: BBS, Bergs Balance Scale; DGI, Dynamic Gait Index; TUG, Timed Up and Go; STS.

Table S2. Correlation between GAITRite parameters and functional balance tests in patients using walkers.

	Singe task				Dual task				Average
	**BBS**	**DGI**	**TUG**	**STS**	**BBS**	**DGI**	**TUG**	**STS**	**(absolute)**
Velocity	0.22	0.47	-0.46	0.07	-0.09	0.06	-0.43	0.59	0.30
Cadence	0.20	0.73	0.25	0.00	0.07	0.73	0.43	-0.19	0.33
Stride length	-0.02	0.22	-0.57	-0.04	-0.07	0.09	-0.61	0.37	0.25
Swing time	0.20	0.60	-0.14	-0.44	0.29	0.15	-0.50	0.41	0.34
Double-support time	-0.20	-0.60	0.14	0.44	-0.12	-0.28	0.43	-0.30	0.31
Stride length variability	-0.52	0.34	0.49	0.17	0.14	0.12	-0.60	0.55	0.37
Swing time viarability	-0.06	-0.31	0.49	-0.09	-0.05	-0.49	0.26	-0.46	0.28

Abbreviations: BBS, Bergs Balance Scale; DGI, Dynamic Gait Index; TUG, Timed Up and Go; STS.

Table S3. Correlation between GAITRite parameters and functional balance tests in patients not using walkers.

	**Singe task**				**Dual task**				**Average**
	**BBS**	**DGI**	**TUG**	**STS**	**BBS**	**DGI**	**TUG**	**STS**	**(absolute)**
Velocity	0.64	0.47	-0.61	0.33	0.75	0.51	-0.69	0.36	0.55
Cadence	0.55	0.39	-0.48	0.37	0.55	0.39	-0.48	0.24	0.43
Stride length	0.62	0.44	-0.60	0.27	0.75	0.50	-0.68	0.33	0.52
Swing time	0.65	0.49	-0.67	0.45	0.76	0.54	-0.74	0.45	0.59
Double-support time	-0.63	-0.42	0.64	-0.40	-0.71	-0.48	0.69	-0.40	0.50
Stride length variability	-0.44	-0.33	0.38	-0.14	-0.57	-0.25	0.46	0.14	0.34
Swing time viarability	-0.52	-0.47	0.46	-0.21	-0.67	-0.57	0.62	-0.35	0.48

Abbreviations: BBS, Bergs Balance Scale; DGI, Dynamic Gait Index; TUG, Timed Up and Go; STS.
