# Supplementary figures and images for: Validity of the GAITRite Walkway Compared to Functional Balance Tests for Fall Risk Assessment in Geriatric Outpatients
Source: Geriatrics (Basel). 2020 Oct 17;5(4):77. doi: 10.3390/geriatrics5040077 (PMC7720110; doi:10.3390/geriatrics5040077)

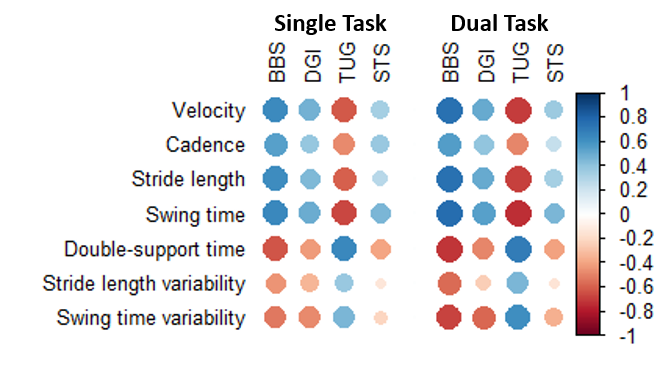

Supplement: Supplementary file 1 [file geriatrics-05-00077-s001.zip › supplementary/Figure S1.tif]
